# Thiolated Silicone Oils as New Components of Protective Creams in the Prevention of Skin Diseases

**DOI:** 10.3390/ma14164723

**Published:** 2021-08-21

**Authors:** Agnieszka Kulawik-Pióro, Anna K. Drabczyk, Joanna Kruk, Magdalena Wróblewska, Katarzyna Winnicka, Justyna Tchórzewska

**Affiliations:** 1 Department of Chemical Engineering and Technology, Institute of Organic Chemistry and Technology, Cracow University of Technology, Warszawska 24, 31-155 Kraków, Poland; anna.drabczyk@pk.edu.pl; 2 Department of Engineering and Machinery for Food Industry, Faculty of Food Technology, University of Agriculture in Kraków, Balicka 122, 30-149 Kraków, Poland; joanna.kruk@urk.edu.pl; 3 Department of Pharmaceutical Technology, Faculty of Pharmacy with the Division of Laboratory Medicine, Medical Univeristy of Białystok, Mickiewicza 2C, 15-222 Białystok, Poland; magdalena.wroblewska@umb.edu.pl (M.W.); katarzyna.winnicka@umb.edu.pl (K.W.); 4 Lubricant Supply Chain, Shell Business Operations Poland, Czerwone Maki 87, 30-392 Kraków, Poland; justyna.legiec95@gmail.com

**Keywords:** barrier creams (BC), thiomers, silicone oil, skin adhesive, skin barrier function, occlusion, protective preparations, prevention of skin diseases

## Abstract

This work investigates the possibility of using thiolated silicone oils as new components in protective creams and their impact on the efficacy of these products. Thiolated silicone oils were synthesized by amide bond formation between primary amino groups of poly17dimethylsiloxane-co-(3-aminopropyl)-methylsiloxane] and carboxylic groups of thiol ligand (3-mercaptopropionic acid) with carbodiimide as a coupling agent. To evaluate and compare the properties of these kinds of thiomers, three different emulsion o/w types were obtained. Emulsion E1 contained methyl silicone oil, E2 poly[dimethylsiloxane-co-(3-aminopropyl)-methylsiloxane], and E3 thiolated silicone oil (silicone-MPA), respectively. Physicochemical properties, including pH, conductivity, droplet size distribution, viscosity, and stability, were assessed. The efficacy of barrier creams in the prevention of occupational skin diseases depends on their mechanical and rheological properties. Thus, the method which imitates the spreadability conditions on the skin and how structure reconstruction takes places was performed. We also investigated textural profile, bioadhesion, protection against water and detergents, and water vapor permeability. Emulsion E3 was characterized by beneficial occlusion, spreadability, and adhesion properties. These features with prolonged residence time on the skin can make designed barrier creams more preferable for consumers.

## 1. Introduction

Skin diseases are the most frequent occupational diseases that have a direct influence on the quality of workers’ lives, cause their long-term absence from work, and also adversely affect their economic situation [1,2,3]. Currently, one of the prevention methods against occupational skin diseases is the use of cosmetic preparations applied to the skin. In accordance with the guidelines [4], these preparations are called occupational skin products and are part of a three-step skin disease prevention program. In order to protect the skin against weak irritants, the preparations are applied before work. However, they cannot replace other skin protection measures, including protective gloves. The basic action of these preparations, i.e., protective creams (PCs), is to make a protective barrier on the skin in the form of a membrane or film when exposed to harmful conditions. This is strictly connected to the composition of the product and adhesion to the skin on which it is applied for a specific, rational period of time, until it wears off or is removed by handwashing [5,6]. 

In the literature, we can find studies [5] confirming the hypothesis proposed by Sadhra, Kurmi, and Mohammed et al. [7] that the lack of effectiveness of barrier creams (protective preparations) is a result of insufficient adhesion to the skin, weak mechanical resistance, and lack of restoration of the structure created on the tissue. These facts were a starting point for us to find new compounds with higher bioadhesion to the skin, longer skin retention time, improved barrier properties, and occlusion ability. If such compounds are introduced to the recipes of creams, it will be possible to obtain effective preparations against occupational skin diseases.

Silicones are known to be used in treatment products and in skin protection preparations against skin diseases such as atopic dermatitis (AD) or irritant contact dermatitis (ICD). Moreover, they are safe, non-comedogenic, hypoallergic, and water-resistant [8,9,10,11]. No interference with stratum corneum lipid organization has been discovered [12]. The protective film created by silicone oils is not oily, does not inhibit the natural skin respiration, and protects against mechanical factors and water evaporation. Moreover, the compounds from this group improve performance of cosmetics by making the skin soft and smooth [13]. Thiolation itself is not a brand new solution either.

Thiomers belong to an innovative class of biomaterials, and they contain sulfhydryl groups (–SH). Thiol groups in polymer chains give rise to significant mucoadhesion properties [14,15,16]. So far, the use of silicone oils to synthesize thiolated derivatives was proposed only Alexandra Partenhauser and Andreas Bernkop-Schnürch’s team. The raw material used in the study was poly[dimethylsiloxane-co-(3-aminopropyl)-methylsiloxane] (silicone oil) with an equivalent of functional groups 4400 Da and viscosity of 100 cSt. The thiomers were obtained by, among other techniques, creating an amide bond between the primary amine group of the silicone oil and the carboxyl group of thiol ligands (3-mercaptopropionic acid (MPA), thioglycolic acid (TGA), 2-mercaptonicotinic acid (MNA)) in the presence of HOBt ester and carbodiimides and by forming an amide bond between the primary amine group of the silicone oil and the carboxyl group of the dimer of the MNA. In the reaction with the dimer, an entirely S-protected silicone oil was obtained, which is used as a second generation mucoadhesive agent [14].

The adhesion of thiomers to mucous membranes, the indications of the presence of free thiol groups in human skin, and the versatile use of silicones in skin applications were the starting point for silicone-MPA and silicone-TGA research [8,17,18]. This research concerned both the determination of the characteristics affecting dermoadhesion for thiolated silicone oil derivatives [8] and an assessment of their potential as dermoadhesive excipients [18].

An ideal agent protecting skin against allergens, irritants, and contact dermatitis must show increased bioadhesion, longer residence time on the skin surface after topical application, water resistance against washing, low water vapor permeability, and the ability to reduce transepidermal water loss (TEWL) [8].

One of the factors influencing the substance’s wash-off resistance is its substantivity. Both studied thiomers (silicone-TGA, silicone-MPA) had an extended residence time on porcine skin of up to 8 h, compared to dimethicone and non-thiolated amino silicone oil, which were detectable up to 4 h. For silicone-TGA, it was 20%, and for silicone-MPA, 40% [19]. The first explanation for extended residence time of thiolated silicone oils can be that hair on the skin’s surface is primarily composed of keratin. This protein has a cleavable disulfide bonds. These new derivatives may interact with those disulfide bridges through interchanging reactions. A keratin-thiolated silicone oil network can be created, and the thiomers attach to the skin surface [8,17,18,20]. Another reason that thiolated silicone oils are adhesive is the fact that interactions between keratin layers, which are the major components in the stratum corneum as the outermost shield of the skin, can occur [8,18]. The conception that disulfide exchange reactions might likely be between thiomers and skin was proposed earlier by Valenta et al. [21]. The authors [8] also showed that at the same level of the number of –SH groups present in thiomers, pK_a_ of the thiol ligand influences the adhesive properties and wash-off resistance. Thus, the MPA ligand with a higher pK_a_ forms intermolecular cross-linked networks via sulfide bond formation between polymer chains, while TGA forms intramolecular networks [22].

The assessment of the skin barrier functions carried out for these thiomers in a test with synthetic urine showed that the silicone-MPA derivative forms an almost impermeable protective film for 3 h (no more than 2.5% leaking dye), and after another 3 h, the percentage of the released quantity of methylene blue is below 25%. For silicone-TGA, an attenuated but still significant protective effect is evident. During the research, the non-thiolated amino silicone oil and dimethicone did not show significant differences in comparison with the control sample [8].

The difference between the thiolated derivatives of silicone oils and their non-thiolated counterparts and dimethicone also lies in their flow properties. Both dimethicone and non-thiolated oil were spread easily. Silicone-MPA was determined to be non-spreadable. Intermediate results were obtained for silicone-TGA. Different values of spreadability can be explained by viscosity differences of analyzed derivatives and cross-linking caused by thiolation. Thiomers may regulate the flow properties of cosmetic products and affect their acceptance by users [8].

Thiolated silicone oil as a potential skin protective agent also showed low water vapor permeability (cumulative water loss < 0.2 g, time 8 h). Cumulative water loss for non-thiolated oil was about 0.5 g (time 8 h), similar to the dimethicone used for comparison. It can be concluded that thiolation not only increases bioadhesion but also lowers water vapor permeability through the created film. An important factor here is the above-mentioned internal cohesiveness due to the formation of disulfide bonds. This obviously results in an interlocked network hindering water penetration [8]. By lowering permeability, thiomers may increase skin moisture and, as a result, its protection. The effect of thiomers on the moisture level was supported by TEWL tests (in vitro with Franz cell setup). Silicone-MPA was significantly more occlusive than paraffin oil. The TGA derivative also showed pronounced reduction of water loss at the start of the measurements. At the end of the experiment, it approached higher values, such as those of non-thiolated silicone oil. Because different results of water vapor permeability were obtained according to the applied ligand, it is possible to select a thiomer adequate for the recipe of the protective agent with a specific purpose [8].

Despite such promising properties, only one study proposal was found in the literature. According to that proposal, only the derivative (silicone-TGA) was analyzed as a dermoadhesive excipient, and it was a hydrogel component. Moreover, it was a comparison study against hydrophilic thiomers [18,20].

In the course of their study, the authors determined the bond strength between modified or unmodified polymers on skin slices from pig ear skin to confirm thiomer-based adhesion to the skin. They carried out tensile studies (total work of adhesion (TWA) and maximum detachment force (MDF) were determined) for unmodified hydrogel formulation, thiolated hydrogel formulation, lipophilic silicone oil, and thiolated silicone formulation. Both bond strength and TWA and MDF were higher for thiomers and formulations, respectively, on their basis compared to their non-thiolated counterparts. Thiolated polymers have a very high affinity for the skin. However, the value of the studied parameters was closely related to the type of thiomer (hydrophilic or lipophilic) and also to the structure of its chain (straight or branched).

The silicone-TGA oil (formulation) analyzed in the studies had significantly lower adhesion properties than hydrophilic thiomers, but still, the adhesion ability was enhanced by a factor of five compared to its non-thiolated counterpart [18]. The differences in the adhesion properties of thiolated silicone oil formulations and hydrophilic thiomer formulations are due to the number of free –SH groups in the derivative obtained. A smaller quantity of free thiol groups results in less binding sites for a covalent bond formulated between the skin and thiomers. For thiolated silicone oils, a chemical modification of amino silicone is necessary for which the theoretical value of –SH groups was 227 μmol/g of the polymer and 216 μmol/g of the substitution grade, respectively. Bioadhesion is influenced by a stable network and may be also affected by the molecular weight. The studied hydrophilic polymers tested had a higher molecular weight than 4400 Da of the amino silicone oil (group equivalent) studied.

The physicochemical form of the studied formulations was also important. The formulation of a thiolated silicone-TGA oil was more fluid in comparison with hydrogels in which hydrophilic thiomers were used. As a result, the silicone-TGA penetrated the skin more easily, but also flowed from the surface of the sample of the evaporated skin, which could affect the results obtained. Therefore, it is not known whether this derivative will behave differently in hydrophobic preparations, where it will be one of the main components.

The following points should be noted.

Thiolated silicone oils are biomaterials with improved skin substantivity, barrier function, appropriate spreadability, and high occlusivity and wash-off resistance. This means that introducing them into the recipe of a protective preparation may increase its residence time on the skin at the application area, thus lowering the frequency of application during the day. Moreover, such formulations can stay on scarred skin or be applied on large surfaces [8,18], which is recommended in the treatment of skin diseases and occupational diseases, where the skin barrier functions are already weaker, and dermatosis is already visible. There is no need to add other adhesive compounds to the recipe, as this limits the recipe to necessary components, reduces manufacturing costs, and minimizes the number of adverse effects, including allergies [8,18].

Thiolated silicone oils may be successfully used in such cases where the barrier function of the skin is weakened as well as to protect the skin which is exposed to moisture-associated damage: maceration [8].

The research results on dermoadhesion properties presented in the literature so far cover only the silicone-TGA derivative in a hydrogel formulation out of lipophilic polymers. There are no studies on the introduction of thiolated silicone oils to the recipes of emulsions.

The aim of our study was to introduce a silicone-MPA derivative to the recipe of protective preparations used in a wet environment and to verify the effect of an added thiomer on the preparation features such as spreadability and efficacy (effect on occlusion, absorbability, washability, adhesion).

## 2. Materials and Methods

### 2.1. Materials

As shown by the literature analysis [8,18], the silicone-MPA derivative showed higher viscosity limited to spreading on the surface, better wash-off resistance, better barrier properties, lower vapor permeability, and higher occlusion ability than the silicone-TGA derivative, while both polymers had the same number of substituted –SH groups. Therefore, to produce a protective preparation for use in wet environments, we used a derivative obtained during the synthesis of poly[dimethylsiloxane-co-(3-aminopropyl)-methylsiloxane] with 3-mercaptopropionic acid (Section 2.2).

The safety of the materials used to obtain protective creams was assessed on the basis of the supplier data, public toxicological data, and the results of cytotoxicity research for silicone-MPA published in the literature [8,19].

Table 1 contains the list of chemical reagents used in the synthesis of the thiolated silicone oil (silicone-MPA), obtaining the barrier creams and performing physicochemical analyses.

### 2.2. Synthesis of Thiolated Silicone Oil

**Figure 1:**
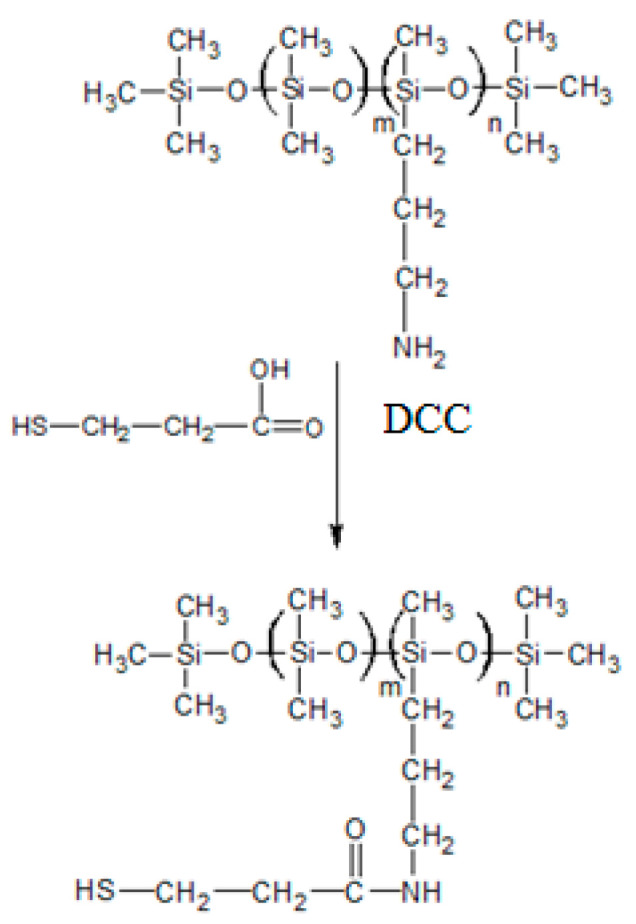
Scheme for thiolation of poly[dimethylsiloxane-co-(3-aminopropyl)-methylsiloxane] in one step synthesis. 3-mercaptopropionic acid was used as thiol ligand and DCC as coupling reagent.

Silicone-MPA was obtained by using a variant of the synthesis in the presence of carbodiimides, without the use of a coupling agent for hydroxybenzotriazole (HOBt), as proposed by Partenhauser et al. [8,19]. The thiolation reaction was carried out by creating an amide bond between the primary amine group of the poly[dimethylsiloxane-co-(3-aminopropyl)methylsiloxane - silicone oil (unmodified) and the carboxyl group of the ligand used. The reaction scheme is shown in Figure 1.

A 1 mmol (1eq) solution of the silicone oil, 2 eq of pyridine and 2 eq DCC at 40 cm^3^ DCM were cooled to the temperature of 0 °C. The droplets of 2 eq MPA dissolved in 2 cm^3^ DMSO were added. Then, the mixture was blended for 1 h at 0 °C. Stirring was continued at room temperature for another 24 h. After this time, purification by filtration of product (thiomer) and a second step of washing with demineralized water were conducted. Procedure was finished when pH of the aqueous phase reached neutral value. The remaining excess of the solvent was evaporated on the evaporator. The thiolated silicone oil was centrifuged twice. The obtained silicone-MPA was kept at 4 °C until further use.

The derived silicone-MPA was checked with infrared spectrophotometry, FT^S−1^65 spectrophotometer (FTIR Biorad, Krefeld, Germany). FT-IR spectra were registered with 128 scans in the wavenumber range from 4000 cm^−1^ to 400 cm^−1^ at resolution of 1 cm^−1^ and at 22 °C. Pure polymer was used.

### 2.3. Determination of Thiol Groups (–SH)

The number of substituted –SH groups in the silicone oil chain was determined according to the methodology with DTDP (4,4′-dithiodipyridine) proposed by Partenhauser et al. [8] and Egwim [23]. The reaction medium was 0.1% *v*/*v* triethanolamine solution. A measurement of 0.03 cm^3^ silicone-MPA was dissolved in 3 cm^3^ of -1ste reaction medium, and 0.05cm^3^ 12 mM DTDP was added. After 5 min, the reaction was completed by adding 0.1 cm^3^ of ice-cold acetic acid to the sample. The absorbance measurement, at a wavelength of 360 nm, was made with spectrophotometer NANOCOLOR UV-vis (Macherey-Nagel, GmbH&Co, KG, Düren, Germany). DMSO was used as background. The number of –SH groups was determined on the basis of the calibration curve of thiol groups determined on the basis of a series of solutions containing growing concentrations of 3-mercaptopropionic acid.

### 2.4. Obtaining Protective Creams

In the course of comparative studies, three o/w emulsions were produced, which differed in the type of the silicone oil used. For this purpose, we used methyl silicone oil POLSIL OM-100 (sample E1), poly[dimethylsiloxane-co-(3-aminopropyl)methylsiloxane (sample E2), and thiolated derivative poly[dimethylsiloxane-co-(3-aminopropyl)methylsiloxane-silicone-MPA (sample E3). The detailed formulation of the protective preparations together with the function of individual ingredients is presented in Table 2.

The components of the individual phases were weighed on the analytical scale. Stearic acid was warmed in a water bath to 80 °C. After melting, PPG-15-Stearyl Ether was added in portions, continuing to mix until a uniform mixture was obtained. When the temperature of the mixture was 70 °C, suitable silicone oil was added in portions and mixing was continued. Then, by maintaining the water phase temperature (previously heated to 70 °C), it was added in small portions to the oil phase by stirring intensively (700 rpm). After 10 min, the cream was removed from the water bath and stirred (500 rpm) until the emulsion reached room temperature. The IKA RW 20 DIGITAL laboratory agitator was used to prepare samples E1–E3. The obtained protective cream was placed in the container and left in a refrigerator for further examination.

### 2.5. Analysis of the Physicochemical Properties of Protective Preparations

#### 2.5.1. pH and Conductivity of Emulsion

pH and conductivity of the emulsion was determined at room temperature (20 ± 2 °C) by placing the pH-meter/conductor measuring electrode of the S47 SevenMulti Mettler Toledo tester (Mettler-Toledo Sp. z o.o., Warsaw, Poland) in the examined sample. The pH/conductivity values were read. The measurement was repeated 3 times. The results presented are the average of the obtained values.

#### 2.5.2. Stability of Emulsion

The stability of the emulsion was assessed in two stages. In the first stage, the sample of the examined emulsion was centrifuged for 10 min at 3500 rpm (919 RFC) (EBA 20 centrifuge, Hettich Zentrifugen, (Andreas Hettich GmbH & Co. KG, Tuttlingen, Germany). After this time, the sample was visually assessed for phase separation. If the emulsion sample did not destabilize in the second stage, accelerated aging tests were performed. For this purpose, the sample of the examined emulsion was placed in an incubator at 40 °C for 24 h and then removed. Its stability was assessed visually. Then, when the sample was stable, it was kept in refrigerator for the next 24 h at 4 °C. The sample was removed again and its stability assessed. The cycle 40 °C/4 °C was repeated 3 times. The samples that had passed 3 cycles were considered stable. The stability testing was conducted on the basis of Cosmetics Europe Guidelines on Stability Testing of Cosmetic Products [24].

#### 2.5.3. Droplet Size Distribution and Microscopic Structure

The obtained emulsions were examined visually and microscopically. The distribution and size of the dispersed phase droplets was observed under the Motic B1 Advanced Series optical microscope (Motic Asia, Hong Kong, China). The photographs of the samples were taken 24 h after they were produced, using 10× and 40× magnification. The smell and consistency of the samples were also assessed.

The distribution of the dispersed phase droplets of the examined emulsions was determined on the Zetasizer Nano ZS Malvern Instruments analyzer (A.P. Instruments Sp. z o. o. Sp. k. Warsaw, Poland). The scattered light was measured an angle of 173 deg. Dilution of tested samples with distilled water (up to 1% wt) was included in procedure to minimize effect of multiple scattering. The measurement temperature was 25 °C and relative humidity—60%.

### 2.6. Rheological Studies

Rheological investigation of obtained emulsions was performed using rotational rheometer RS6000 Haake (Thermo Scientific, Karlsruhe, Germany). A cone-plate measuring system (cone’s parameters: d = 35 mm, 2°) was applied. The measuring gap was 0.105 mm. For each emulsion, experiments were performed three times. The measurement temperature was 25 °C. The applied measurement procedure is described in detail in [5].

#### 2.6.1. Study of Viscoelastic Properties

During measurements, the values of G’ and G” moduli in the deformation function (γ) (frequency 10 Hz) were measured. The measurement consisted of two steps. Step 1: heating the sample for 300 s (up to 25.0 °C ± 0.1 °C), relaxation of sample (60 s), and controlled deformation in the range of 0.0001–10.0. Step 2: sample relaxation (60 s at the set temperature) and controlled deformation in the range of 0.0001–10.0. Sample relaxation in step 2 followed controlled deformation in step 1.

#### 2.6.2. Flow Curves

Flow curves were obtained by hysteresis loop quality test. Shear stress and normal stress were determined in the mode of controlled increasing (curve up) and decreasing (curve down) shear rate 0 s^−1^—100.0 s^−1^ and 100.0 s^−1^—0 s^−1^, respectively. The start of the measurement was preceded by heating the sample (300 s, up to 25.0 ± 0.1 °C) and reset of the normal force. 

The quantity of energy dissipated by the sample (E) was calculated based on Equation (1) [5].

E = P ⋅ Δt ⋅ V(1)

where P is surface area between the flow curves up and down calculated with trapezoidal method, Pa⋅s^−1^; t is measurement of time, s; and V is volume of sample, 4⋅10^−6^ m^3^.

#### 2.6.3. Determination of Yield Stress

To determine the value of yield stress, analyzed samples were loaded with stress, which increased linearly over time (300 s^−1^ time of measurement). During this procedure the changes of deformation were observed. The maximum value of applied stress was 0.1 Pa. The yield stress (τ_0_) is defined as a point (value of stress), where two fitting straight lines intersect in the (log(τ)–log(γ)) coordinate system.

### 2.7. Texture Analysis

**Figure 2:**
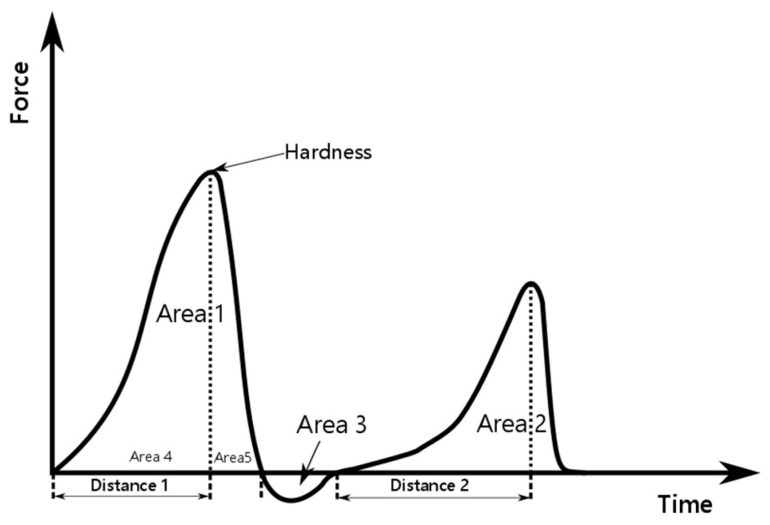
The interpretation of the results from the texture profile analysis test: adhesiveness: Area 3, cohesiveness: Area 2/Area 1, compressibility: Area 1, hardness: maximal force obtained after first deformation [5].

The texture profile analysis test (TPA) with a 75% percent deformation of the specimen height was performed. The texturometer (a single-column strength machine) SHIMAZU EZ Test EZ-LX (Shimadzu Scientific Instruments, Kyoto, Japan), equipped with an acrylic probe, with a diameter of 20 mm and length of 40 mm, was applied. The probe shift speed was 2 mm⋅s^−1^. Five samples of each emulsion type were subjected to texturometric testing at 25 °C. The same sample volume (40·10^−6^ m^3^) and measuring container (diameter = 38 mm) were provided. The applied measurement procedure is described in detail in [5]. The determination and calculation of texture parameters using TPA test are described in Figure 2 [5,25].

### 2.8. Efficacy

Effectiveness assessment of the protective preparations produced for application in a wet environment, their occlusion ability, absorbability, water washability, and bioadhesion properties was determined ex vivo on the skin model of hairless mice [5,6,26,27]. As a reference substance (for the occlusion ability), Vaseline was used. This compound has an occlusive effect. It can be used in a standardized test procedure as a standard reference substance in evaluation of protective or barrier cream against water-soluble and water-insoluble irritants [28].

#### 2.8.1. Parallel-Plate Method: Spreadability 

To evaluate spreadability of the samples E1–E3 (at 25 ± 0.5 °C and 32 ± 0.5 °C), the parallel-plate method was applied [5,6,26].

The surface coated (S_i_, cm^2^) by the analyzed sample under a given load was measured. The plates (top and bottom) of normalized weight 86 ± 1 g and dimensions 10 cm × 10 cm were used.

One gram of examined emulsion was put on the center of the bottom plate and covered with the top plate. After 10 min, the spreading area (spots) of the emulsion was measured clockwise three times with mechanical polar planimeter HA-317 E (GEBRUEDER HAFF GMBH, Pfronten, Germany). Final results were calculated as arithmetic mean from measurements [29].

#### 2.8.2. Payne Cup Occlusivity Test

The measure of occlusivity of cosmetic preparations is vapor transmission rate (Equation (2)), determined in vitro by the Payne cup method, based on ASTM Standard E96 [30]. The higher the transmission rate, the more the occlusivity decreases.

WVT = 240⋅Δm/A(2)

where WVT is water vapor transmission rate, g⋅m^−2^⋅d^−1^, and A is cup surface, 10 cm^2^.

The experiment was carried out at 21 ± 0.5 °C and constant humidity with Payne Permeability Cup Elcometer 5100 (Elcomter, SciTeex Sp. z.o.o, Warsaw, Poland), filled with distilled water at 22 °C. The collagen foil was a semi-permeable membrane, on which 0.1 g of sample was applied. The measuring chamber was closed. The water loss was measured by weight after 15 min for a period of 2 h. The experiments were conducted in three repetitions for each emulsion. Final results were calculated as arithmetic mean from measurements [29].

The occlusivity of the preparations was also determined for 6 h. The measurements were made after 2, 4, and 6 h, respectively.

#### 2.8.3. Absorbability

Laboratory methods developed by Central Institute for Labour Protection–National Research Institute (CIOP-PIB) and modified by our team [26,27,29] were applied to resistance analysis of protective emulsion against water, aqueous detergent solutions, acids, and bases. The applied measurement procedure is described in detail in [29]. In this gravimetric method, the absorbability of samples coated with a layer of the protective preparation was evaluated.

In the first step in the test, the squares (60 mm side) were cut from the collagen film and then weighed on analytical scale (RADWAG, Cracow, Poland) with 0.0001 g accuracy. In the analyses, only these squares, whose weights did not differ by more than 10%, may be used. Seven squares were prepared for each sample including a zero sample.

A total of 0.2 g of analyzed sample (E1–E3) was spread on squares and left for 20 min to dry and to achieve constant weight. The zero sample was squares not covered with emulsion. After 20 min all squares were weighed on an analytical scale with 0.0001 g accuracy.

In the second step, 200 μl of distilled water or 1% soap water solution was added with a pipette in three places on each sample, including the zero sample. Samples were left for 15 min. 

To remove the remaining droplets on the surface of collagen foil (squares), the samples were shaken three times. After that, they were weighed.

Absorbability of a protective cream is calculated from Equation (3):

(3) $$N=\frac{M_{2}-M_{1}}{M_{1}}\cdot 100\%$$

where *M*_1_ is the weight of the sample before the water or aqueous detergent solution is added, g; *M*_2_ is the weight of the sample after the water or aqueous detergent solution has been added, after specific time, g.

For calculated values of absorbability, authors defined the arithmetic mean, standard deviation, and the coefficient of variation for the formula. If the coefficient of variation was greater than 25%, an additional three measurements were performed, with recalculation of arithmetic mean, standard deviation, and coefficient of variation.

Evaluation of final resistance of protection against water and detergent solutions was established on the resistance criteria by CIOP-PIB [26,27,29].

#### 2.8.4. Water Washability

An effective protective preparation for the skin used in an aqueous environment applied to the skin should form a protective layer (barrier). When the protective cream is more miscible with detergents, this protective film can be removed rapidly and more precisely. To determine if our emulsions are resistant against washing, the water washability test was performed. The method suggested by Treffel et al. [31] was applied. 

A total of 0.5 cm^3^ of the sample was placed on the back of the hand. Then, it was washed using cold running water for 15 s by hand rubbing. After 15 s, if there was negligible amount of emulsion remaining on the skin, then the sample could be rated as water washable [29]. The applied measurement procedure is described in detail in [29].

#### 2.8.5. Ex Vivo Bioadhesive Properties Using Hairless Mice Skin as Adhesive Layer

Evaluation of bioadhesiveness was performed using TA.XT.Plus Texture Analyser (Stable Micro Systems, Godalming, UK) and hairless mice skin as the adhesive layer. The skin was acquired from Cby.Cg-Foxn1nu/J hairless mice intended for collection of organs (Centre of Experimental Medicine, Medical University of Białystok, Białystok, Poland). The approval of the Local Ethical Committee on Animal Testing was not required. After freezing (−20 °C), skin samples were stored for a maximum of 4 weeks.

Just before experiment, skin was defrosted and cut into 5 mm diameter pieces. After that, skin samples were thawed for 30 min in physiological saline solution (0.9% NaCl, at 25 °C ± 0.5 °C). Next, mice skin was connected to the lower end of a cylindrical probe using a cyanoacrylate glue. Analyzed samples (0.5 g) were placed below the probe. The experiment was conducted at 32 °C ± 0.5 °C (water bath) to simulate skin temperature. The experimental parameters of the process were applied force of 0.5 N, contact time of 120 s, pre-test speed of 0.5 mm∙s^−1^, test speed of 0.1 mm∙s^−1^, and post-test of 0.1 mm∙s^−1^. These parameters were determined by preliminary tests. The bioadhesive properties were expressed as the maximum detachment force (F_max_) and the work of adhesion (W_ad_, µJ). Measurements were performed 6 and 7 times, respectively. From obtained results, the arithmetic mean was calculated.

The work of adhesion (W_ad_) was calculated according to Equation (4):

W_ad_ = A · 0.1 · 1000 (4)

where A is area under the force versus distance curve, which is multiplied by 0.1 as the conversion time measurement to distance (the sampler was raised at 0.1 mm·s^−1^) and then multiplied by 1000 in order to express the result in units of work, µJ.

Since the results of the characteristics tests (number of –SH groups, number of –NH_2_ groups, reaction effectiveness) and the studies of the physicochemical properties of thiolated derivatives on the basis of poly[dimethylsiloxane-co-(3-aminopropyl)methylsiloxane were published by Partenhauser’s team [8,14,19], in this work, the detailed experimental studies only involved the products obtained: the emulsions intended to protect the skin in a wet environment. For comparative purposes and to ensure correct discussion of the results for the oils used (POLSIL OM100, silicone-oil, silicone-MPA), the spreadability and the occlusivity were determined using the test methodology for samples of protective creams. For the obtained silicone-MPA derivative, the number of –SH groups and the thiolation reaction efficiency were determined in accordance with the methodology described in Section 2.3.

#### 2.8.6. Statistical Analysis

Whenever applicable, the gathered results were presented as mean ± standard deviation (SD).

The analyses of variance (ANOVA) were carried out to check differences between F_max_ and W_ad_ (work of adhesion) of the protective creams (emulsions E1–E3). A value of *p* < 0.05 was considered statistically significant. Tukey’s HSD post hoc comparisons were performed when the significant difference was 95%.

## 3. Results

### 3.1. Synthesis of the Thiolated Silicone Oil 

**Figure 3:**
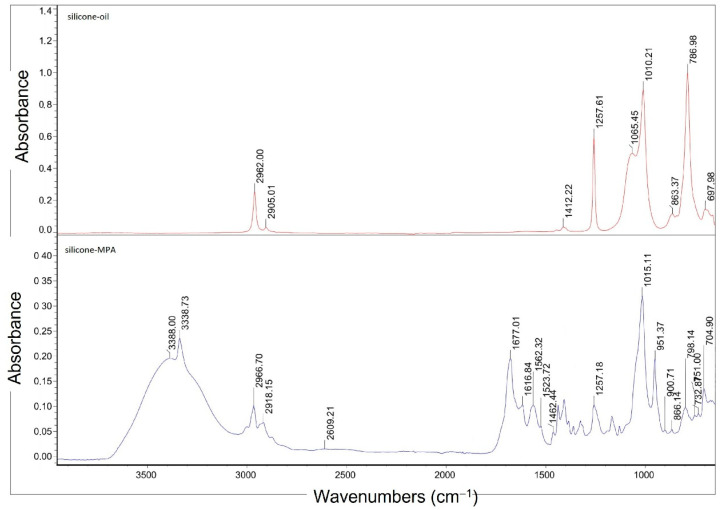
FTIR spectra of unmodified silicone oil (red) and thiolated derivative—silicone-MPA (blue).

The IR spectrum analysis of the created silicone-MPA confirmed the reaction and obtaining the desired product. On the spectrum of the thiolated derivative (Figure 3), individual stretching bands derived from amide N–H bonds are visible at a wave number of 3400 cm^−1^. The stretching vibrations of the C=O carbonyl group in the amide I band can be found for the wave number of 1677 cm^−1^. The signal between 1575 and 1460 cm^−1^ is the result of N–H bending in addition to C–N stretching (amide II band), while the bands of –SH groups are in the range of the wave number of 2600 cm^−1^. In comparison with the IR silicone oil spectrum, in the silicone-MPA spectrum, the bands at 1260 cm^−1^, characteristic for the CH_3_–Si–O–CH_3_ dimethyl group and trimethyl substituent CH_3_–(CH_3_–Si–O)–CH_3_ in the polymer chain, are less intensive. Additionally, the intensity at the wave number of 2961 cm^−1^ also decreased for the band responsible for the polysiloxane chain length. In the silicone-MPA spectrum at the 1015 cm^−1^ wave number, a doublet of Si–O–Si stretching vibrations occurred that end at ca. 800 cm^−1^, where the vibrations of the –CH_3_ group and Si–C stretching vibrations can be observed [32,33].

The effectiveness of the thiolation reaction was 46 ± 18%, which corresponds with 104 ± 41 μmol/g of –SH groups (the maximum number of groups possible for substitution 227 μmol/g of the polymer) in the thiolated oil. Our results are in good agreement with Partenhauser’s [19].

### 3.2. Characteristics of Emulsion

The study results for the physicochemical properties of the tested emulsions are shown in Table 3.

**Figure 4:**
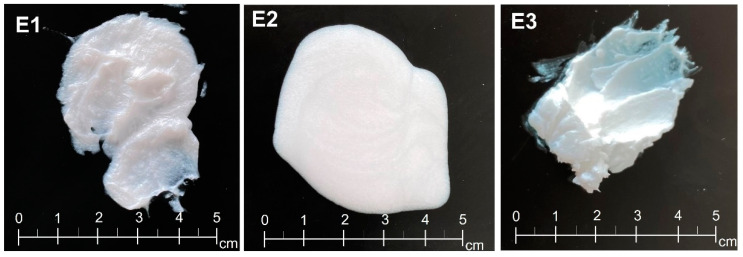
Visual evaluation of consistency of 1 g samples E1–E3.

The oils used in the recipe differed in color (POLSIL OM 100—slightly yellow, silicone oil—colorless, silicone-MPA—milky yellow), but this did not affect the visual properties of the obtained emulsions. All the obtained protective emulsions were white. However, the use of a different type of silicone oil results in different odor, consistency (Figure 4), pH value, and conductivity.

Out of the used silicone oils, the thiolated derivative (silicone-MPA) had an unpleasant odor (characteristic for thiols), which persisted despite introduction into the E3 emulsion recipe. In the E3 sample, dichloromethane used in silicone-MPA synthesis was not perceptible. This was most likely caused by its low boiling point (40 °C) and the fact that emulsification was conducted at a temperature higher than the boiling point of this solvent. Nevertheless, it should be emphasized that protective creams intended for use in wet environments are classified as cosmetic products and are subject to Regulation No 1223/2009 of the European Parliament and of the Council. According to Annex III: list of substances which cosmetic products must not contain except subject to the restrictions laid down, item 7, an unintended quantity of dichloromethane in a cosmetic product is permissible as contamination which, subject to good manufacturing practice for technological reasons, is unavoidable. The concentration of dichloromethane may not exceed 0.2% wt. [34].

Silicone oils are not always compatible with emulsifiers, which results in difficulties in obtaining stable preparations. In the literature [35,36,37,38,39,40], different emulsifier systems were studied. Higher HLB values are preferred for stabilization of silicone emulsions [40]. We used triethanolamine stearate with HLB 20 [41] and PEG-8 stearate with HLB 11.1 [42], which allowed us to obtain stable samples regardless of the silicon oil used. Long acid chains can stabilize the lipophilic silicone molecule inside the micelle due to hydrophobic interactions [40].

The emulsions obtained were of the o/w type, which was confirmed by a conductivity study. All the analyzed samples had good electrical conductivity due to the water phase rich in electrolytes derived from, e.g., the dissociation of TEA-stearate anion emulsifier. When the –SH group polymer chain was introduced, it reduced conductivity by ca. 15%.

pH of the skin with occupational dermatoses may differ from the typical pH range of 5–6 of healthy skin [43]. However, the pH of protective creams should be as close as possible to these values to avoid skin irritation, impairment of the lipid barrier, and the integrity of the stratum corneum [43,44]. Out of the obtained samples, only E1 met this condition. The samples (Table 3) with silicone oil (unmodified) and silicone-MPA had significantly higher pH than the E1 sample. The TEA-stearate emulsifier used in the recipe has a pH of 8.8–9.2. If the excess of stearic acid is used, the pH has values that do not adversely affect the condition of the skin. Therefore, in the case of the E2 and E3 samples, a higher pH value was a result of the presence of –NH_2_ groups for E2 and –NH_2_ and –SH for E3 in the silicone oil chain. The amine group has alkaline properties. The nitrogen atom present with a free electron pair may fulfil an alkaline role in accordance with Lewis’s definition. In the presence of water, it is ionized to NH_3_^+^ and creates OH^-^ ions in the system. The –SH group does not undergo protonation.

The partial substitution (substitution rate of 45.8%) of –NH_2_ groups with –SH groups in poly[dimethylsiloxane-co-(3-aminopropyl)methylsiloxane lowers the pH value of the sample. In the case of complete substitution, it may not be necessary to introduce an additional pH regulator into the E3 formulation. As shown by Metha [45], pH changes the ionization of function groups in a silicone molecule, thus affecting its stability or the type of emulsion that modified silicone oils can stabilize. However, due to the relatively low polarization of the amine group, amino silicones do not have good emulsification capacity, and modified silicone oils with an ionic nature stabilize the w/o emulsions [45].

The consistency of the product is one of its most important features. It is the result of the cohesiveness, density, and viscosity of the product. The consistency of the product is closely related to its recipe and, in particular, to the presence of substances such as waxes, emollients, thickening substances, and rheological property modifiers [5,6,46,47,48]. The consistency is also a feature that makes the products attractive to consumers. It is worth adding that it affects the effectiveness of the action of creams applied to the skin [46,49]. Due to their intended purpose, protective preparations used in wet environments contain hydrophobic compounds. These materials have high melting temperatures and/or high viscosity, which can cause difficult application and a heavy consistency. Preparations with a light consistency have good application and distribution properties, and they are absorbed quickly. Preparations with a heavier consistency are more effective in covering the skin surface, and, once adhering to it, they form a protective barrier [50]. Out of the obtained samples of protective preparations, E3 had the heaviest, compact consistency (Figure 4). This was most likely the result of the high viscosity of the silicone-MPA used. The E1 sample based on methyl silicone oil had a soft consistency, resembling ointment. The appearance of the E2 emulsion resembled body lotion, as it was liquid. The differences in the consistencies of the samples reflect the results of our studies of rheological properties, spreadability, and efficacy. 

**Figure 5:**
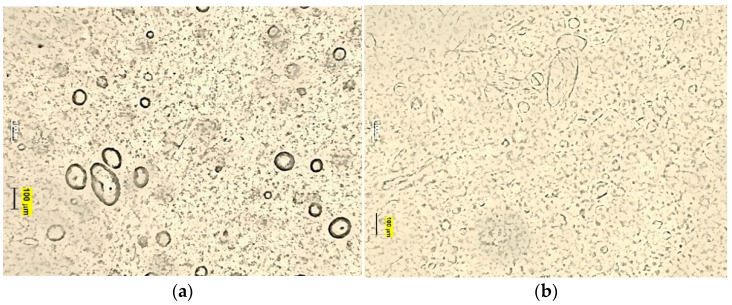
Microscopic image of a cream with methyl silicone oil, sample E1, magnification (**a**) 10×, (**b**) 40×.

**Figure 6:**
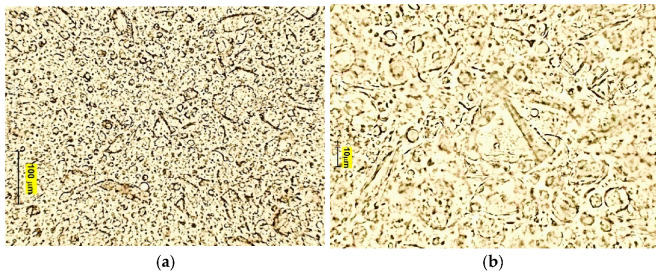
Microscopic image of a cream with silicone oil, sample E2, magnification (**a**) 10×, (**b**) 40×.

**Figure 7:**
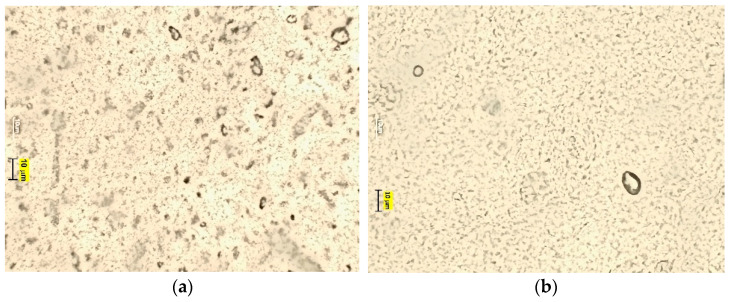
Microscopic image of cream with silicone-MPA, sample E3, magnification (**a**) 10×, (**b**) 40×.

Figure 5, Figure 6 and Figure 7 show microscopic images of the E1–E3 samples studied.

The microscopic images of the studied protective creams and the PDI value (Table 4) of the samples clearly indicate that the samples obtained are polydisperse systems. The highest PDI value was found in the E1 sample, and the smallest in the E3. The modification of the amino silicone oil chain by introducing thiol groups resulted in increased viscosity and thus the viscosity of the dispersed phase. Due to an efficient disulfide cross-linking, the enhanced viscoelastic properties of thiolate silicone oil were observed. As the viscosity of the silicone oil used increases, the emulsion sample is characterized by droplets with a larger diameter—Z-Ave (d.nm) increased. The high viscosity of the oil phase made it difficult to break it down into smaller droplets. This is in good agreement with results published by Nazir et al. [39], yet contrary to works by Partenhauser [40]. There, the self-emulsifying drug delivery system (containing stearic acid, Span 80 Bri O 10, butanol) obtained by the authors did not present statistically significant differences in the droplet size for systems containing amino silicone oils and silicone-MPA. However, this may be influenced by the degree of substitution of the polymer chain with –SH groups. In the works, the degree was 97%. The droplet size of the dispersed phase in the E3 emulsion could be also influenced by protonation of NH_2_ groups and interaction of the positively charged amine group and the micelles of the surfactant [40]. The increased droplet size of the dispersed phase did not negatively influence the stability of the studied samples.

### 3.3. Rheological Properties of Emulsions

#### 3.3.1. Study of Viscoelastic Properties

**Figure 8:**
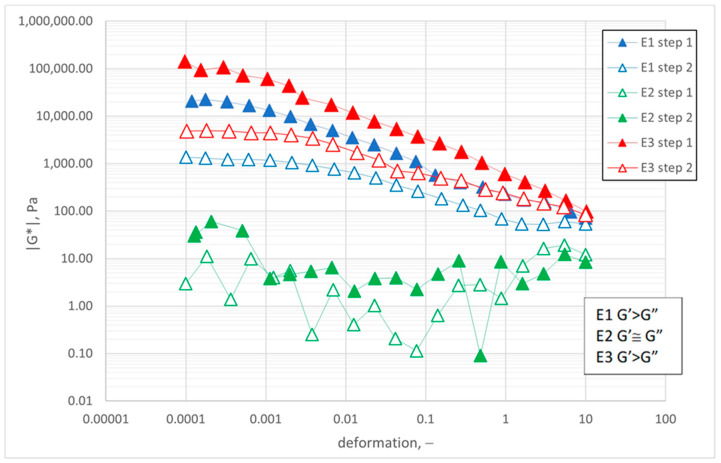
|G*| module against variable deformation for samples E1–E3.

The aim of the applied measurement method was to simulate the conditions associated with spreading protective preparations on the skin and to check if the structure reconstruction occurred when shearing disappeared [5]. In step 1, the change in the values of G’ and G” in conditions of variable deformation was analyzed. Step 2 was the study of restoration of the system structure. In the case of the analyzed protective creams, it was shown that emulsions E1 and E3 were systems with viscoelastic properties. On the other hand, emulsion E2 showed purely viscous rheological properties (Figure 8). In the case of E1 and E3 emulsions, the classic curves of G’ and G” were not observed in the form of clearly isolated linear viscoelastic regions (LVR) and non-linear viscoelastic regions. However, sample E1, in a small deformation range (below 0.001), showed properties characteristic for a linear region. It is shown by parallel lines of G’ and G” curves with regard to the axis showing deformation changes. In the first step of the experiment (increasing deformation value), the highest |G*| values were noted for E3. This means that this system showed stronger viscoelastic properties than E1. In the whole range of the deformation, the |G*| module values decreased for emulsions E1 and E3 (by 3 decades); for the highest deformation value |G*|, emulsion E1 reached the lowest value (71.04 Pa) in comparison to emulsion E3 (98.13 Pa). This behavior shows that the structure of sample E3 was characterized by higher resistance to deformation than E1. It was shown in another measurement of the changes in the modulus of elasticity in the function of the applied deformation (step 2) that the structure of samples E1 and E3 was weakened. As a result, lower values of the |G*| modulus were observed for the whole deformation range. The starting values of this parameter were not achieved despite sample relaxation. This phenomenon shows that the structure of the studied emulsion was changed. Lowering the applied deformation did not enable the reconstruction of its structure.

#### 3.3.2. Flow Curves

Depending on the physicochemical form of a cosmetic preparation, its high apparent viscosity can be considered an advantage by users, as it proves, in their opinion, the very good quality of the product, and it is associated with a high concentration of active substances in the recipe. Yet, especially in the case of emulsions, it can also be a significant disadvantage of the product. The excessive viscosity of the preparation makes it difficult to be distributed, which discourages users from applying it regularly and frequently. However, by modifying the apparent viscosity of the protective emulsions, the residence time of the cosmetic on the skin can be prolonged, and the production of an occlusive layer, spreadability ease, and rinsing can be changed. It has also been proven [51] that formulations with high viscosities may remain to a greater extent in skin furrows. 

Applying protective creams on the skin is associated with various physical operations, which directly correspond to certain values of the shear rate. As indicated in [5,52,53], these procedures contain in shear rate ranges between 0.01 and 10 s^−1^, draining under gravity, and in shear rate ranges between 10 and 100 s^−1^, cream spooning and pouring. Cream rubbing corresponds to a shear rate around 1000 s^−1^. Table 5 shows the apparent viscosity values depending on the shear rate. The largest apparent viscosity was determined for sample E3 with silicone-MPA, then for samples E1 and E2. These results indicate different behaviors of the analyzed samples depending on the shear rate, which may affect their application to the skin, including, in particular, the spreadability of creams. In addition, silicone-MPA, due to its intermolecular cross-linking ability, is a raw material with a much higher viscosity than methyl silicone oil, and it can be added to formulations for topical use as a modifier of rheological properties.

**Figure 9:**
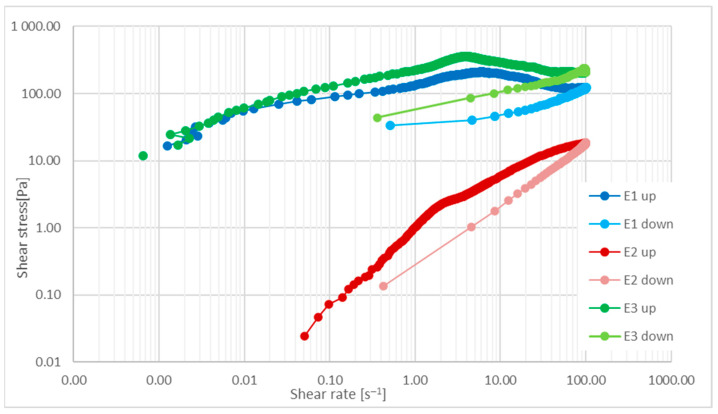
Flow curves of samples E1–E3.

The spreadability of the cosmetic on the skin is also influenced by the value of the yield stress or its lack. The protective emulsions produced are non-Newtonian shear thinning systems with yield stress. Sample E3 had the highest yield stress value. This value was almost 2 times higher than for sample E1 containing silicone oil. All tested samples shown a hysteresis loop, i.e., a difference in the relation course τ(γ) measured in the conditions of increasing and decreasing shear rate (Figure 9). In none of the analyzed cases does hysteresis disappear, which means that none of the received preparations rebuilds the system structure after the disappearance of shear. In this case, this is an undesirable feature. The calculated value of the energy dispersed by the studied creams can be ranked in the following order: sample E3 > sample E1 > sample E2. It follows that the smallest changes to the emulsion structure due to the shear rate used are observed for cream E2, while the largest changes are for cream E3.

According to Lukic [54], hand creams should also be pleasant to use when rubbing them in. Thus, they should have a low yield stress and maximum viscosity (η_0_ apparent viscosity at γ = 0 s^−1^), whereas G’ must be at least one order of magnitude larger than G”. The last condition, i.e., G’ > G”, is met only by samples E1 and E3. Sample E3 also has the highest viscosity among the tested emulsions, but it does not meet the third criterion of low yield stress (Table 5).

### 3.4. Texture Profile

Texture measurements have shown that the tested emulsions are different in terms of textural properties (Table 5). This means that the type of the silicone oil used in the recipe, especially its viscosity, has an important influence on the textural characteristics of protective preparations. Moreover, the chemical modification applied—partial substitution of –NH_2_ groups with –SH groups in the amino silicone oil chain—leads to obtaining an emulsion (E3) with different texture characteristics than the starting emulsion (E2).

The hardness value is the peak force that occurs during the first compression. Samples with higher hardness are more difficult to remove from the container or spread on the skin. The highest hardness was found in a sample containing silicone-MPA (E3), and sample E2 had the smallest hardness. The differences in the value of this parameter were large, as the value for preparation E3 was approximately 4 times higher than for E1 and almost 50 times higher than for E2. In addition to the presence of silicone-MPA in the formula, the high hardness value of sample E3 was also influenced by stearic acid.

Compressibility can be explained as the work needed to deform the product during the first compression of probe. The compressibility expresses the ease with which the emulsion is taken from the package and spread over the skin. This value should be as low as possible [5,55,56]. The compressibility value was the highest for sample E3 (22.423 ± 0.895 N·s), and an over 5 times lower value was noted for E1 (4.307 ± 0.194 N·s) and the lowest for E2 (0.285 ± 0.008 N·s).

The highest compression value obtained for sample E3 is consistent with the fact that the sample was the most difficult to be distributed, and this may cause discontinuity of the layer formed on the skin.

Cohesiveness and adhesion are texture parameters that are associated with the distribution of cosmetic products on the skin surface [5]. Cohesiveness is defined as a direct function of the work necessary to overbear the internal forces of the sample. The low cohesiveness of the preparation demonstrates the existence of weak influences shaping its structure. When cohesiveness increases, the resistance of preparations to mechanical actions decreases. These observations were confirmed with rheological studies. Sample E2 had the highest cohesiveness (0.936 ± 0.039), and sample E3 had the lowest (0.497 ± 0.024). This is the only parameter for which emulsion E2 obtained the highest value and indicates little effect of the compression on its consistency compared to other systems. The resulting values of the texture differentiators described above indicate significant differences in the consistency of the studied systems, which was already evident during the visual evaluation of the finished emulsions (Figure 4) and which is reflected in their application on the skin.

In the case of the analyzed systems, adhesion is particularly important, as it is helpful in predicting the local residence time. This parameter is defined as the work necessary to overcome the attraction force between the studied sample and the adhesive model layer [57]. Emulsions E1 and E3 show adhesiveness, while emulsion E2 does not. Moreover, sample E3 exhibits the highest adhesiveness (indicating high interaction with the sensor surface) while at the same time having low cohesiveness. The lack of adhesiveness when creating or developing emulsions with barrier properties is a negative phenomenon, disqualifying the system for such a purpose. The highest adhesiveness value was noted for the E3 system, which indicates a positive effect of silicone-MPA on the adhesiveness of the received emulsion. The observed differences between sample E1 and E3 for apparent viscosity, yield stress, viscoelastic, and textural properties are related to the viscosity of the oil used and the intermolecular cross-linking effects found in the thiol derivative.

### 3.5. Efficacy of Protective Products

**Figure 10:**
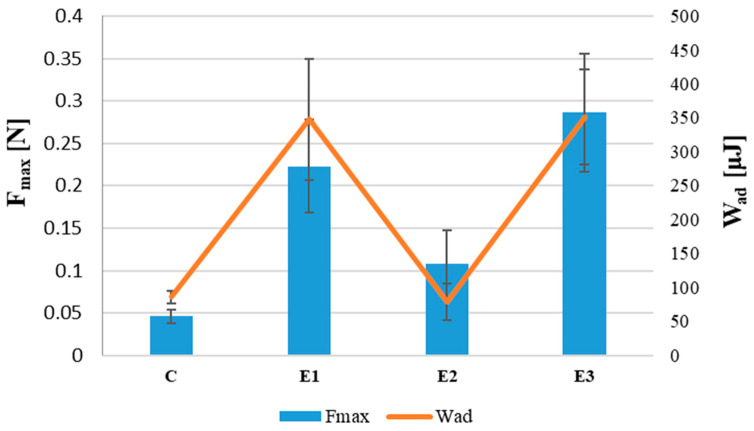
The maximum detachment force F_max_ and the work of adhesion W_ad_ for samples E1–E3 and negative control (cellulose paper, sample C).

To evaluate the usefulness of the studied skin protection products, they were tested for washability, water resistance, spreadability, occlusion ability, and ex vivo bioadhesion properties on the skin model of hairless mice. The results are shown in Table 6 and Figure 10.

The preparations produced are o/w emulsions. Despite the introduction of hydrophobic substances such as stearic acid into the preparations, under the assumed test conditions, they were partially washable (samples E1 and E3) or completely washable (sample E2). After washing off, samples E1 and E3 left a greasy deposit on the skin. In addition, the skin after removal of sample E3 had the smell of thiol. 

The type of silicone oil used significantly influenced the results of the occlusion ability of the preparations and their spreadability, which was in line with our assumption. The lowest degree of water vapor permeability and spreadability was determined for the preparation containing silicone-MPA. On the other hand, the resulting protective emulsions had low (samples E1 and E3) and medium (sample E2) water resistance and, respectively, good (sample E1) and medium resistance (sample E2 and E3) against detergents. 

Sample E3 had the highest values of F-_Max_ and W_Ad_. The lowest values of these parameters were obtained by sample E2.

## 4. Discussion

Protective creams (PCs) act in a purely physical way, as they create a barrier on the skin that hinders the penetration of harmful agents. Therefore, the PCs best suited to protect the skin in a wet environment and against hydrophilic irritants are mainly preparations such as lipophilic ointments or w/o emulsions. With regard to lipophilic irritants, o/w emulsions are recommended [4].

According to [4], the rule that the lipo- or hydrophilic character of PCs limits their effectiveness against irritants and allergens both lipo- or hydrophilic is considered oversimplistic. When it comes to the effect on the epidermis, PCs can act in three ways. These are immediate, intermediate, and delayed effects. The immediate effect is occlusion, which occurs due to addition of a lipid mixture to the skin surface. In the intermediate effect, the lipid mixture is added to intercellular spaces. The delayed effect concerns supplying lipids to the epidermal cells, helping these to enhance the natural lipid production and lipid release. This restores the natural barrier function [58,59]. To achieve the delayed effect, one of the solutions is to enrich the cream recipe with lipids that are chemically related to the physiological content of the intercellular domain of the horny layer in a topical formulation (i.e., a mixture of diglycerides and triglycerides, free fatty acids, waxes, cholesterol, squalene, or ceramides). As a consequence, the preparation has protective, regenerative, and care effects. However, the preparations with a higher proportion of care and emollient ingredients have a weaker protective effect [59,60].

In order to be as effective as possible, it would be best if the preparation showed a dualistic mode of action [4]. This means that PCs should combine the action of hydrophilic compounds (i.e., propylene glycol, glycerin, sorbitol) and lipophilic compounds (i.e., stearic acid, dimethylpolysiloxane). We developed a recipe for a protective preparation, which was an o/w emulsion: a system with rapid penetration deep into the skin with immediate formation of a thin protective film on the skin surface [61]. It contained stearic acid, emollients, glycerin, and thiolated silicone oil. The oil could be successfully used in cases where the skin barrier function is compromised and also in protecting the skin against moisture-associated damage, i.e., maceration.

To evaluate the quality and usefulness of the preparation containing silicone-MPA, we determined its spreadability, washability, resistance to water and detergents, occlusion ability, and adhesion under ex vivo conditions. The results were compared with preparations containing methyl silicone oil or amino silicon oil used for thiolated derivative synthesis, respectively.

### 4.1. Spreadability

Our perception of consistency depends on mechanical parameters such as plasticity, viscosity, elasticity, and other rheological properties such as yield stress and resulting spreadability [46,62,63]. Good spreadability of the preparation on the skin is one of the most important components building the image of the product on the industrial market. Cosmetic preparations which spread well on the surface of the skin are more likely to be used than products which are difficult to apply, especially in the case of prevention of occupational skin diseases, where it is necessary to apply a new dose repeatedly during the workday [4]. Consistency is a sensory feature, and it is determined on the measurement of rheological, textural, and distributional properties.

Cosmetic preparations with silicones are appreciated by consumers because they spread easily, have lower tackiness, and are silkier and more slippery in touch compared to preparations without silicones [10,64]. The obtained protective cream with thiolated silicone oil did not have any of these characteristics.

Spreadability of the silicone oils used for the production of samples E1–E3 was consistent with the data from the literature [8]. Methyl silicone oil had spreadability similar to amino silicone oil, but it was more than twice as high as silicone-MPA. Although amino silicone oil and methyl silicone oil spread easily, sample E1 had almost 4 times a lower spreadability than sample E2. This may be connected to the fact that sample E2 was very thin, spilled over the surface, and had the lowest viscosity among the emulsions tested (Table 5). The most compact consistency and highest viscosity was found in sample E3, which resulted in the lowest spreadability. These observations are consistent with the performed textural characteristics, as the highest values of the parameters, i.e., hardness, compressibility, and adhesiveness, were obtained for this emulsion from among the emulsions tested (Table 5). It can therefore be concluded that if silicone-MPA oil is introduced into the emulsion, it does not lose its properties, such as the ability to create intermolecular cross-linking disulfide bonds. For all the samples, it was observed that the area of the resulting stain increases when the measured temperature increases as well. Protective emulsion samples at 32 °C corresponding to the temperature of the epidermis spread better, which is advisable due to their application to the skin.

In our opinion, spreadability was also one of the factors influencing the washability of products and their resistance against water and detergents.

### 4.2. Washability and Absorbability

The observed partial washability of the obtained preparations was due to the fact that they were o/w emulsions and they contained hydrophilic emulsifiers such as Cithrol PEG-8 stearate and TEA-stearate. TEA-stearate is also used in recipes of cleansing products for skin and hair. This compound helps water to mix with dirt and, as a result, wash it away easily.

The washability of the products influenced their absorbability, especially when detergent was used. The poorer detergent resistance of preparations with silicone-MPA compared to a sample containing methyl silicone acid may be due to the fact that sample E3 had the highest viscosity and highest yield stress and the least spreadability of all the samples. These characteristics caused weak interaction of the produced film with the membrane, which was also the result of the inability of this preparation to rebuild the structure. Therefore, the layer produced on the collagen membrane (collagen film) could fail to dry under the set time conditions. Additionally, the surface on which the detergent solution was applied could have been incompletely covered. In the case of emulsion E2, the average resistance against both water and detergents may be due to the fact that the preparation had a low viscosity and low yield stress. Moreover, it may be the result of its thin consistency, as it quickly spilled on the surface of the collagen membrane and dried. Nevertheless, the results of the detergent resistance tests are contrary to the results published by Partenhauser [8] and the fact that silicones are known to form an easily distributed hydrophobic film.

The explanation for these differences may be first due to the fact that the studies by Partenhasuer et al. concerned the assessment of the properties of pure thiolated oils, whose degree of substitution was approximately 93%. Secondly, the study was conducted on pig skin, where, as the authors noted themselves, thiolated silicone oil could interact with disulfide bonds of keratin layers, which are the main component of stratum corneum [8,21], and, moreover, the high substantivity and adhesion depended on the pKa of the acid ligand used. The value of pKa for MPA was 4.35, and the polymer chain substitution rate in our substrate was 46%, which caused a low amount of thiol anions in the system. The amount of thiol anions was also limited by the pH of the medium (water or detergent solution) acting on the sample when applied to the collagen film. When the amount of thiol anions was limited, the more efficient formation of disulfide bonds was between separate molecules of thiolated silicone oil. This eventually leads to stronger inner cohesiveness. In the case of stronger thiol acids (e.g., TGA), the dominant mechanism was the formation of intramolecular cross-linked networks via disulfide bond formation between polymer chains [8]. As the pH of the sample increases (sample E3 had pH equal to 8.2), –SH groups should oxidize, and disulfide bonds also should be formed quicker. On the other hand, if stearic acid with pKa = 4.75 close to MPA is present in the recipe, it can interfere with this process.

### 4.3. Occlusion Ability

**Figure 11:**
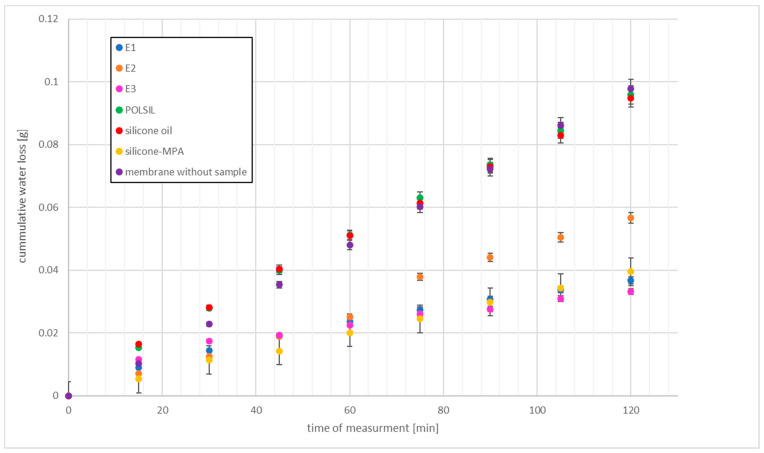
Change of cumulative water loss for analyzed oils, samples E1-E3, membrane without sample after 2 h in 15-min intervals, established by Payne Cup method.

The degree of water vapor permeability expressed as cumulative water loss was determined with the Payne Cup method within 2 h for samples of E1–E3 for the oils used. The results are shown in Figure 11.

The silicone oils used in the recipe differed in their occlusion ability. After two hours, the water vapor permeability of methyl silicone oil and amino silicone oil was similar and was 1150.8 and 1153.6 g/m^2^⋅d, respectively. Silicone-MPA oil (P = 497 g/m^2^⋅d) showed 2.3 times lower water vapor permeability than other oils. These results are in agreement with Partenhauser’s data [8]. The value of water vapor permeability for silicone-MPA was more than 22 times higher than for Vaseline, whose water vapor permeability rate after 2 h was 22.4 g/m^2^⋅d.

Methyl silicone oil and unmodified (amino silicone) oil are not occlusive, as they create a permeable layer on the skin. This property should not be limited if they are introduced into the recipe of cosmetic emulsions [65,66]. The observed decreased water vapor permeability in the emulsions produced on their basis, i.e., E1 and E2, respectively, is therefore caused by stearic acid in their recipe. The share of stearic acid in the recipe is the same, so these values should be similar. Therefore, the difference in the results follows from consistency, viscosity, spreadability, and adhesion of the preparation.

The lowest water vapor permeability for emulsion E3 is the result of higher cohesiveness due to disulfide bonding, which causes an interlocked network that hinders water penetration. Thiolated silicone oil does not lose its properties during the emulsification. In this case, the film produced on the skin surface can improve the barrier functions of the skin, protect against moisture loss (it increases the content of water in the skin), and, above all, inhibit the loss of water in physiological evaporation processes [67]. Moreover, due to its chemical structure, this film does not bond with liquid crystalline structures of stratum corneum lipids [12,66,68].

**Figure 12:**
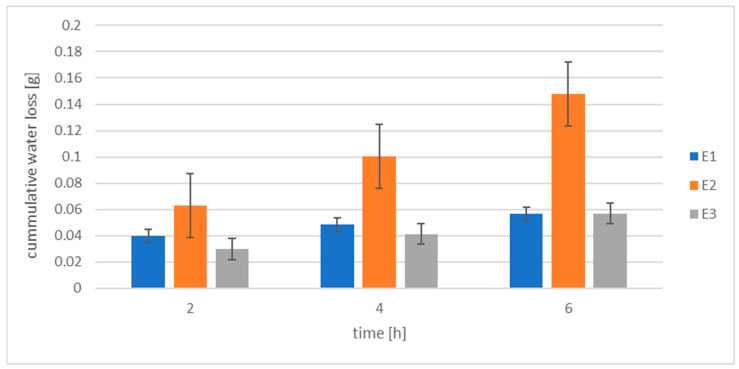
Change of cumulative water loss for analyzed samples E1–E3 after 2, 4, and 6 h, established by Payne Cup method.

Protective preparations intended for the skin are applied for a period declared by the manufacturer. On average, this time is from 2 to 4 h; however, a new layer has to be applied after each hand wash. If the formulation contains a derivative which exhibits occlusion over a longer period, this will cause the preparation to have an increased barrier function, skin hydration, and protection. To test this hypothesis, we determined the occlusion of the emulsions tested up to 6 h. The results are shown in Figure 12.

The advantage of emulsion E3 containing thiolated silicone oil over E1 containing methyl silicone oil is visible within 4 h. In addition, water vapor permeability of sample E3 after 2 h is almost 17 times higher than for Vaseline, whereas for sample E1 the difference is 21 times. After 6 h, the water loss for samples E3 and E1 is the same. This is confirmed by the data found in the literature [66]: the occlusive properties of silicones are determined by the structure of the chain and its degree of substitution. In order to increase the barrier properties of silicones, silicone oils with higher viscosity are used. Linear silicones are highly permeable even if they have high molecular weight. Therefore, subjecting silicones to thiolation makes it possible to obtain raw materials with reinforced occlusivity [8], and unlike polydimethylsiloxanes, it does not require introduction into silicone backbone long-chain hydrocarbons (C18 and longer).

The commonly observed water resistance improving the protective function of creams or lotions with silicones is mistakenly interpreted as being the result of their high occlusion. Firstly, preparations applied to a large body area should have high water vapor permeability, and therefore, silicone-based preparations (i.e., dimethicone) used in daily care can be water resistant and have a protective role; however, they do not have to be occlusive. In the case of the examined protective preparations strictly aimed at effective protection in the water environment during work, silicone-MPA is, first and foremost, the substance increasing the occlusion of the preparation and subsequently responsible for its hydrophobicity. Amino silicone oil, on the other hand, makes sample E2 more hydrophilic and less occlusive [69].

### 4.4. Adhesion

For the protective preparation to remain for a certain time on the skin surface, it must be substantive. Due to this feature, the protective layer on the skin cannot be easily rinsed or rubbed [10]. In the case of silicones, depending on the type of substituent attached to alkyl siloxane, its physical properties can be manipulated, or hydrophobicity, viscosity, and mechanical strength can be altered. Substituents with low volume cause easy rotation in the siloxane chain (no steric hindrance). Thanks to the substituent-forced orientation of the “outside” structure, it is possible to synthesize siloxanes with the desired adhesion [67].

As the texture profile study showed, sample E3 was 4 times more adhesive than sample E1. However, the ex vivo study of the bioadhesive properties of the emulsion did not show statistically significant differences between these samples. The statistically significant differences were determined between samples E1 and E2 and E2 and E3 (Table 7).

The results show that formulation E2 has the worst bioadhesion properties and that samples E1 and E3 are good and comparable. The highest values of parameters F_Max_ and W_Ad_ for sample E3 are associated with the highest values (among the protective preparations analyzed) in rheological characteristics, such as viscosity, yield stress, and textural characteristics, such as adhesion, hardness, and cohesiveness. According to Partenhauser [8,17,18,20,21], the reason for the extended residence time of the pure silicone-MPA compared to dimethicone and non-thiolated amino silicon oil is interactions between keratin layers, which are the major components in the stratum corneum as the most outward barrier of the skin, or interaction with the keratin present in the hair covering the skin. The absence of significant differences in the adhesion properties of formulations E3 and E1 is therefore due to the number of –SH free groups in the obtained derivative. A lower amount of free thiol groups means fewer binding sites for covalent bonds formulated between the thiolated polymer and the skin. These results are indications that, in order to ensure adhesion to the skin, it is necessary to chemically modify the polymer chain, which, as a result, leads to a product with a certain degree of substitution. Obtaining a stable network of –SH group interactions may also depend on the molecular weight of the polymer.

### 4.5. Limitations of Use of Thiolated Silicone Oils in Protective Preparations. Stability Assessment

In 2015, more than 50% of present skin care cosmetics contained a minimum of one silicone [10,70]. The reason is that silicones are safe cosmetic raw materials and well tolerated by the skin, with low surface tension and good wetting properties. Caused by a high molecular weight, these compounds are not absorbed deeply into the skin. They are resistant to physical and chemical factors, in particular high temperature, oxidation, and fungal or bacterial activity. When they are introduced into the cosmetic recipe, they protect against water loss, as they increase water content in stratum corneum and limit water loss in physiological evaporation by creating a protective film that does not limit respiration of the skin [10]. They soften and smooth the skin [67]. Their properties are related to their relatively open and flexible chemical structure [10,66]. Their advantages also include odorlessness and miscibility with many substances, such as vegetable oils, fatty acid esters, or alcohols [66,67].

Silicones with an amine functional group have a positive charge at the end of the polymer chain. Their area of application is mainly hair cosmetics, as these compounds are most effective in adsorption to the hair surface due to the strong polar interaction of hair proteins with an amino group [71,72].

Despite the data found in the literature concerning the great potential of thiolated silicon oils as bioadhesive substances, they have various shortcomings such as a cationic nature and limited stability against oxidation [32]. Polycations, despite their versatile and widespread use as drug delivery systems, are known to be hemotoxic via interacting with a cell membrane since overall polyanionic barriers disturb the membrane structure and function. Therefore, both types of amino functions, the charge density, and the arrangement of the cationic charges have meaning [32,73,74]. Another aspect that limits safety and therefore the use of thiolated silicone oils is the fact that DCM is used for their synthesis. Additionally, it should be noted that various substances have to be added to the recipe to mask the smell of a thiolated derivative.

**Figure 13:**
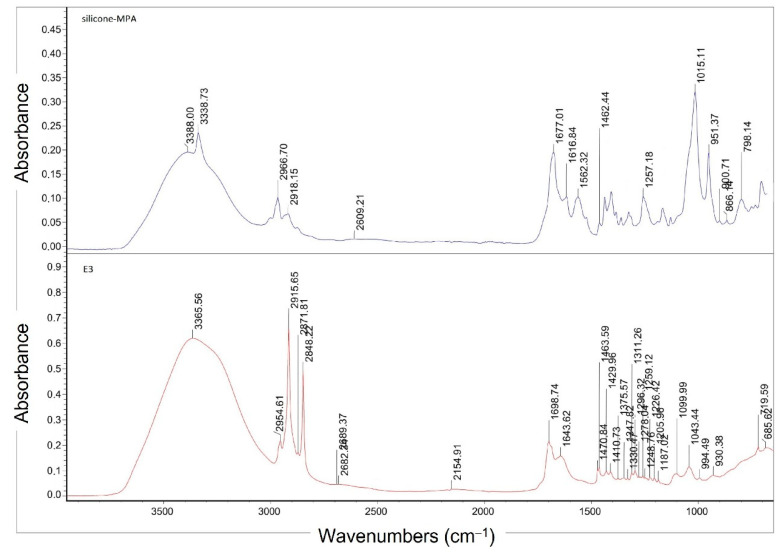
FTIR spectra of silicone-MPA (blue) and sample E3 (red).

At this stage of the study, the IR spectrum of this formulation was made for the initial verification of the durability of our formulation containing a thiolated derivative (Figure 13). The spectrum was compared with the spectrum of the thiolated silicone oil used [75]. In the IR spectrum of sample E3, a band is visible at a wave number of 2960 cm^−1^, which is responsible for the length of the polysiloxane chain and a doublet of Si–O–Si stretching vibrations at a wave number of 1040 cm^−1^. Stretching bands derived from N–H amide bonds at a wave number of 3400 cm^−1^ in the E3 emulsion sample coincide with the range of bands derived from the water contained in the protective cream sample. The E3 emulsion sample also shows peaks characteristic of the silicone-MPA used, i.e., at a wave number of around 1670 cm^−1^ from the stretching vibrations of the C=O carbonyl group in the amide (amide I band) and the band from the –SH groups (with a wave number of about 2600 cm^−1^), indicating that the oil does not degrade when it is added to the recipe. This was proved by the results of studies on the physicochemical properties and effectiveness of the E3 preparation. The 1500–1650 cm^−1^ area seen in sample E3 is characteristic for N–H deforming vibrations. The wide band of 660–910 cm^−1^ may come from N–H wagging vibrations. In both cases, the signal may come from both the non-substituted –NH_2_ groups in the silicone-MPA and triethanolamine, which could have not been neutralized. 

## 5. Conclusions

In these studies, we assessed the potential of using silicone-MPA as a substitute for methyl silicone oil in the formulations of protective preparations intended for use in wet environments. A good protective preparation used for the prevention of occupational skin diseases should have such characteristics as increased bioadhesion, prolonged retention time on the skin, improved barrier properties, and occlusion ability.

When a silicone thiomer in the recipe of a protective cream is used instead of methyl silicone oil, improved characteristics are achieved, such as occlusion ability, spreadability, and adhesion (designated texture parameter). However, properties such as smell, pH formulation, and resistance to detergents deteriorate. In both cases, i.e., for the E1 and E3 emulsions, we obtained stable samples, which were partially washable and non-water-resistant with comparable bioadhesion.

The best occlusion ability (period of 4 h) and adhesion (in both in vivo and ex vivo tests), combined with longer skin contact time during rubbing (the preparation does not spill over the surface), may result in better comfort of use for consumers. In such a case, they do not have to frequently apply other portions of the preparation. Nevertheless, the observed inability to restore the structure during simulated viscoelastic tests gives reason to conclude that silicone thiomers are not universal raw materials for any formulation.

## Figures and Tables

**Table 1:** Materials used in investigation.

Trademark	Abbreviation Used at Work	Producer
poly[dimethylsiloxane-co-(3-aminopropyl)methylsiloxane with a functional group equivalent of 4400 Da, viscosity 100 cSt	silicone oil	Sigma Aldrich
3-merkaptopropionic acid	MPA	Sigma Aldrich
N,N′-dicyclohexylcarbodiimide	DCC	Sigma Aldrich
dichloromethane	DCM	POCH
pyridine	−	POCH
dimethyl sulfoxide	DMSO	POCH
triethylamine	−	POCH
Aldrithiol-4	DTDP	Sigma Aldrich
chloroform	−	POCH
neat acetic acid	−	POCH
stearic acid	−	Chempur
POLSIL OM 100	methyl silicone oil	Silikony Polskie
glycerin	−	POCH
Arlamol™ PS15	−	Croda Inc.
Crodamol™ PMP	−	Croda Inc.
Cithrol™ 4MS	−	Croda Inc.
sodium benzoate	−	POCH

**Table 2:** Protective cream formulation with silicone oil/thiolated silicone oil intended for use in a wet environment. Oil phase: A, water phase: B.

Phase	Ingredient	Function in Recipe	Concentration of Components (% Mass)		
			E1	E2	E3
A	stearic acid	emulsifier in combination with triethanolamine, barrier, and lubricating substance	24.0	24.0	24.0
A	methyl silicone oil	creates a protective, occlusive film on the surface which prevent water loss	10.0	−	−
A	silicone oil		−	10.0	−
A	silicone-MPA		−	−	10.0
B	Glycerin	moisturizing substance	8.5	8.5	8.5
B	Triethanolamine	with stearic acid creates emulsifier, pH regulator	1.2	1.2	1.2
A	PPG-15-Stearyl Ether	emollient	1.0	1.0	1.0
B	PPG-2-Myristyl Ether Propionate	emollient conditioning agent	1.0	1.0	1.0
B	PEG-8 Stearate	humectant, rheology modifier, emulsifier	1.0	1.0	1.0
B	Sodium Benzoate	preservative	0.2	0.2	0.2
B	Deionized water	solvent	53.1	53.1	53.1

**Table 3:** Physicochemical properties of samples E1–E3, + stable sample, − unstable sample.

Sample	Visual Evaluation	pH	Stability	Conductivity mV
E1	white, characteristic smell of stearic acid, soft, ointment-like consistency,	6.8 ± 0.1	+	152 ± 0
E2	white, slight smell of stearic acid, thin product, lotion-like consistency	8.5 ± 0.0	+	158 ± 0
E3	white with a characteristic unpleasant odor from the thiolated derivative, semi-solid consistency	8.2 ± 0.0	+	135 ± 0

**Table 4:** Particle size and polydispersity index (PDI) of samples E1–E3.

Sample	Z-Ave (d.nm)	PDI
E1	781 ± 56	0.734 ± 0.008
E2	662 ± 39	0.586 ± 0.017
E3	946 ± 53	0.504 ± 0.009

**Table 5:** Basic rheological properties for samples E1–E3 and textural profile.

Sample		E1	E2	E3
Basic rheological properties	$\dot{\gamma },\;s^{-1}$	**Apparent Viscosity, Pas**		
	1.0	133.00 ± 6.73	1.02 ± 0.05	214.70 ± 12.81
	10.0	18.99 ± 11.41	0.59 ± 0.04	28.22 ± 1.72
	50.0	1.22 ± 0.07	0.30 ± 0.02	4.41 ± 0.27
	100.0	1.22 ± 0.07	0.18 ± 0.01	2.09 ± 0.12
	**Yield stress** $\tau_{0}$ **, Pa**			
		138.3 ± 15.0	1.4 ± 0.3	226.3 ± 12.0
	**Energy dissipated *E*, J**			
		5.24 ± 0.76	0.54 ± 0.05	9.40 ± 0.93
Texture profile	**Texture parameters**			
	Hardness, N	0.866 ± 0.036	0.075 ± 0.003	3.726 ± 0.185
	Compressibility, N⋅s	4.307 ± 0.194	0.285 ± 0.008	22.423 ± 0.895
	Adhesiveness, N·s	3.687 ± 0.116	−	12.803 ± 0.526
	Cohesiveness, -	0.741 ± 0.029	0.936 ± 0.039	0.497 ± 0.024

**Table 6:** Parameters determining the efficacy of samples E1–E3.

Sample	Washability	Resistance to Water		Resistance to Detergents		Spreadability 25 °C/32 °C cm^2^	Occlusion Ability g/m^2^⋅d
		N_av_ [%]	Assessment	N_av_ [%]	Assessment		
E1	partially washed off, a greasy deposit remains on the skin	35.07 ± 1.70	low resistance	11.64 ± 0.90	good resistance	10.4 ± 0.4 18.8 ± 0.6	478.8 ± 13.9
E2	washed off	18.74 ± 0.89	medium resistance	28.38 ± 1.47	medium resistance	40.7 ± 0.2 78.5 ± 2.0	680.2 ± 15.2
E3	partially washed off, a greasy deposit and the smell of thiol remains on the skin	37.16 ± 1.50	low resistance	29.57 ± 0.37	medium resistance	4.7 ± 0.5 7.5 ± 0.1	378.6 ± 12.5

**Table 7:** One-way ANOVA results.

F_max_					
Groups	Count	Sum	Mean	Variance	Post hoc
E1	7	1.563	0.223	0.003	a
E2	7	0.758	0.108	0.002	b
E3	7	2.002	0.286	0.005	and
Source of Variation	SS	df	MS	F	*p*-value
Between Groups	0.1137	2	0.057	18.161	0.00005
Within Groups	0.0563	18	0.003		
Total	0.1700	20			
**W_ad_**					
**Groups**	**Count**	**Sum**	**Mean**	**Variance**	**Post hoc**
E1	6	2085	347.5	7998.8	and
E2	6	551.2	78.7	713.8	b
E3	6	2108	351.3	4805.4	a
					
Source of Variation	SS	df	MS	F	*p*-value
Between Groups	323,829.0	2	161,914.5	37.927	0.0000008
Within Groups	68,304.4	16	4269.0		
Total	392,133.4	18			

## Data Availability

The authors confirm that the data supporting the findings of this study are available within the article.
